# When Ought Appeals to Cease?

**Published:** 1890-06-14

**Authors:** 


### Passing Topics.

### WHEN OUGHT APPEALS TO CEASE?

The issue of appeals is, in the case of many charities, a never-ending process. It is found by experience that, notwithstanding the opinions of some determined but misinformed people, the value of the work performed, no matter how manifest, is of itself an insufficient pleader, and that no sooner are more direct appeals stopped than supplies languish if they do not altogether cease. So far from subscriptions being influenced by the quality of the work done, it is well known, and has been often remarked, that very inferior institutions may obtain by their importunity what better organisations lack when less adroitly puffed.

But it is not on the general subject of appeals that we are inclined to moralise. We fear it goes without saying that a voluntary system of maintenance will ever mean much solicitation, a great deal of expense in the collection of funds, and not a little regrettable competition for favours between the several institutions, good, bad, and indifferent. What we more particularly want to consider is how far continued appeals are justified when the stress of poverty has abated, when, it may be, some large windfall has been obtained, or when surplus funds, aggregating year by year, have placed the finances in a prosperous condition. Under such circumstances is it any longer a right and seemly proceeding to put forward pleas of impecuniosity, to threaten the curtailment of work, or to announce that the institution lies gasping in the toils of debt?

Or if we forbear to inquire into their morality, let us ask whether these exaggerations are expedient? We greatly suspect it is because there is a general acquaintance with the fact that the end is too often held to justify the means that legitimate appeals fail to secure adequate attention. The cry of "wolf" is so persistent that nobody heeds it. The benevolent public, grown familiar with prophecies of trouble, retains its composure unruffled in view of every threatened calamity, and turns from the pathos of the situation to question its truth. It was Bulwer's Mr. Graves who grew callous to political terrors after having seen, on paper, "eighteen crises, six annihilations of agriculture and commerce, four overthrows of the Church, and three last, final, awful and irremediable destructions of the entire constitution." Similarly, the feelings of the charitable are first shocked and ultimately blunted by the repetition of painful narratives of impending disaster. No doubt appeals of the more aggressive types are usually issued on behalf of societies whose disappearance might well be regarded with a satisfaction which after events rarely afford the opportunity to indulge in; but, on the other hand, there have been recent examples of persistent and importunate begging by really respectable institutions which involved a *suppressio veri* certainly, and we fear something more. It would be difficult to lay down any rule designed to limit freedom of action, even were there any way of enforcing it, and, moreover, special help under some circumstances may be quite rightly allocated to a particular purpose distinct from the ordinary maintenance, thus leaving the daily necessities unrelieved. But it may be suggested to hospital authorities that when their institution has been fortunate enough to obtain a grand prize in the lottery, it would be a graceful concession to the proprieties if they stood aside awhile from the scramble for bread in which their less fortunate brethren are engaged, and that such a course would tend to their ultimate benefit by inspiring the benevolent with that respect for appeals which it would be absurd to deny is now somewhat grudgingly bestowed, because the lesson has been so thoroughly learned that it is not always merited.

Agricola.

